# Genetic Alterations in Oxidant and Anti-Oxidant Enzymes in the Vascular System

**DOI:** 10.3389/fcvm.2018.00107

**Published:** 2018-08-09

**Authors:** Maan A. Awad, Sarah R. Aldosari, M. Ruhul Abid

**Affiliations:** Division of Cardiothoracic Surgery, Department of Surgery, Cardiovascular Research Center, Rhode Island Hospital, Brown University Alpert Medical School, Providence, RI, United States

**Keywords:** reactive oxygen species, cardiovascular disease, coronary endothelium, animal model, oxidant enzymes, antioxidant enzymes

## Abstract

Cardiovascular diseases (CVD) are one of the prime causes of mortality worldwide. Experimental animal models have become a valuable tool to investigate and further advance our knowledge on etiology, pathophysiology and intervention. They also provide a great opportunity to understand the contribution of different genes and effector molecules in the pathogenesis and development of diseases at the sub-cellular levels. High levels of reactive oxygen species (ROS) have been associated with the progression of CVD such as ischemic heart disease (IHD), myocardial infarction, hypertension, atherosclerosis, aortic aneurysm, aortic dissection and others. On the contrary, low levels of antioxidants were associated with exacerbated cardiovascular event. Major focus of this review is on vascular pathogenesis that leads to CVD, with special emphasis on the roles of oxidant/antioxidant enzymes in health and disease progression in vascular cells including vascular endothelium. The major oxidant enzymes that have been implicated with the progression of CVD include NADPH Oxidase, nitric oxide synthase, monoamine oxidase, and xanthine oxidoreductase. The major antioxidant enzymes that have been attributed to normalizing the levels of oxidative stress include superoxide dismutases, catalase and glutathione peroxidases (GPx), and thioredoxin. Cardiovascular phenotypes of major oxidants and antioxidants knockout and transgenic animal models are discussed here.

## Introduction

Cardiovascular diseases (CVD) are the leading cause of morbidity and mortality in the world. Reactive oxygen species (ROS) are believed to play major roles in the pathogenesis and development of CVD including ischemic heart disease and myocardial infarction. This review will focus on vascular pathogenesis leading to the development of CVD. Major emphasis will be given to the roles of oxidant/antioxidant enzymes in the vascular cells including vascular endothelium. The knockout and transgenic animal models of major oxidant/antioxidant enzymes affecting ROS levels in the vasculature and CVD progression are excellent experimental tools to further our understanding of CVD.

Studies have suggested a pivotal role of ROS in various physiological conditions as well as in a wide range of diseases including CVD (1–5). Oxidative stress results from a disrupted balance between ROS production and activity of antioxidant enzymes. Several enzymes, including superoxide dismutase (SOD), glutathione peroxidases (GPx) and thioredoxin act as scavengers to reduce the levels of ROS. In contrast, oxidases including nicotinic adenine dinucleotide phosphate (NADPH) oxidase, nitric oxide synthase (NOS), monoxides and xanthine oxidoreductase (XO) increase oxidant levels in the cardiovascular system (CVS). Several studies have illustrated the roles of ROS in CVD using animal models.

In the current review, we will present several transgenic and knockout animal models for genes encoding major oxidant and antioxidant enzymes and their cardiovascular phenotypes. We will discuss specific phenotypic changes associated with the manipulated expression of these genes in the vascular cells. For the convenience of the readership, we have included two tables, one with the major oxidant and antioxidant knockout (KO) models and the resulting cardiovascular phenotypes (Table 1), and the other with transgenic animal models and their cardiovascular phenotypes (Table 2).

**Table 1:** Knockout oxidant and antioxidant models and their effects on cardiovascular system.

**Effector**	**Tissue/cell (model)**	**Effects and phenotypes**	**References**
NOX1	Total KO (Nox1^y/−^)	**Reduction of neointimal hyperplasia after vascular injury** (1) Inhibition of cell proliferation in the sitting of injury-induced neointimal formation (2) Reduction in apoptosis after vascular injury (3) Reduction in fibronectin accumulation (4) Phosphorylation of Coiflin leading to impaired migration	(6)
	Total KO (NOX1-deficient)	**Regulation of blood pressure and vascular response to Ang-II** (1) In Ang-II-induced, increase 4-fold in NOX2 (2) In Ang-II-induced, minor increase in ROS (3) Decrease in systemic blood pressure upon Ang-II infusion (4) Attenuation of the hypertrophic middle area upon Ang-II infusion (5) Attenuation of the increased distance between elastic fibers and ECM area	(7)
		**Involvement of NOX1 in Ang-II-induced aortic dissection** (1) Significant reduction in the number of mice who had sudden death compared to the wild-type (2) Blood pressure elevation similar in both the presence and absence of NOX1 in a norepinephrine treatment (3) No aortic dissection in norepinephrine-treated mice (4) Massive increase in TIMP1 gene expression compared to Ang-II-induced wild-type	(8)
	Total KO (ApoE^−/−^/Nox1^−/y^)	**NOX1 deficiency decreased the area of atherosclerosis** (1) Decreased ROS production (2) Decreased the number of macrophages in the atherosclerotic lesion	(9)
NOX2	Total KO (Nox2^−/y^/ApoE^−/−^)	**Lack of NOX2 was associated with decrease in the area of atherosclerosis** (1) Decrease superoxide production (2) Increase NO bioavailability	(10)
	Total KO (Nox2^−/−^)	**Reduction of neointimal hyperplasia after vascular injury** (1) Decrease neointimal thickening (2) Decrease cellular proliferation (3) Alter inflammatory cellular infiltration	(11)
gp91^phox^	Total KO (gp91^phox−/−^)	**Role of gp91**^phox^ **in the Regulation of basal blood pressure and pressure-independent vascular hypertrophy to Ang-II** (1) Reduction in basal systolic blood pressure (2) No significant difference in the increased basal systolic blood pressure after Ang-II infusion between KO mouse and wild-type (3) No increase in superoxide anion levels in Ang-II-treated model (4) No increase in aortic medial area (5) No increase in aortic medial area upon Ang-II infusion (6) The presence of gp91^phox^ in the endothelium and adventitia in the wild-type with increase in both superoxide anion levels and aortic medial area upon Ang-II infusion	(12)
		**Ang-II-induced cardiac hypertrophy is dependent on gp91-containing NADPH oxidase** (1) Loss of gp91 mRNA expression (2) Abolition of NADPH oxidase activity, and thus ROS production upon Ang-II infusion (3) Lower basal systolic blood pressure without Ang-II infusion (4) Attenuation of heart/body weight ratio and ANF and beta- MHC mRNA expression (cardiac hypertrophy markers) with Ang-II treatment (5) Decrease in collagen content with Ang-II infusion	(13)
p47^phox^	Total KO (p47^phox−/−^)	**Coronary vasodilatation requires NADPH oxidase-derived ROS** (1) Reduction in NADPH activity, and therefore reduction in ROS level (2) Reduction in P13k-Akt-eNOS, NO production, and VEGF-induced vasodilatation	(14)
		**Pivotal role of NADPH oxidase and p47**^phox^ **in Ang-II-mediated hypertension** (1) No increase in vascular ${\text{O}}_{2}^{-\cdot }$ production after Ang-II treatment (2) Blunted increase in blood pressure response to Ang-II (3) Loss of p47^phox^ expression in aortic endothelium (4) Similar expression of TA1 receptor in p47^phox−/−^ and wild-type	(15)
	Total KO (ApoE^−/−^/p47^phox−/−^)	**Lack of p47**^phox^ **decreased the size and area of the atherosclerotic lesion** (1) Decrease proliferation of Vascular smooth muscle cells	(16)
		(2) Decrease ROS production (3) CD44 expression was decrease in the atherosclerotic lesion
	(ApoE^−/−^/p47phox^−/−^) and (ApoE^−/−^) BMT was made to investigate the contribution of the vascular wall cells and the monocytes/macrophages to the development of atherosclerosis in relation to their expression of p47phox-containing NADPH oxidase	Both monocytes/macrophages and the vascular wall cells contributed significantly to the development of atherosclerosis, which was evident from the significant reduction in the lesion size following the exclusion of the NADPH oxidase activity from any of these two tissues	(17)
eNOS	Total KO (apoE^−/−^/eNOS^−/−^)	**Lack of eNOS activity was associated with increase in the area of atherosclerotic lesions, and lead to the development of other vascular complications** (1) Increase L-E interaction (2) Increase the expression of VCAM-1 by endothelial cells and smooth muscle cells (3) Increase mononuclear infiltration into the plaque lesion (4) Decrease NO level (5) Decrease superoxide production (6) Development of vascular complications including aortic aneurysm and aortic dissection (7) Development of distal coronary arteriosclerosis and perivascular and endomyocardial fibrosis	(18, 19)
iNOS	Total KO (ApoE/iNOS-dKO)	**iNOS deficiency was associated with a decrease in the total area of atherosclerosis** (1) Deacreased plasma lipoperoxide level (2) iNOS deficiency ameliorated the oxLDL-induced inhibition of foam cell migration	(20–22)
nNOS	Total KO (nNOS^−/−^) and (ApoE/nNOS-dKO)	**nNOS deficiency was associated with accelerated neointimal formation and increased total area of the atherosclerotic lesion**	(23, 24)
MAO-A	MAO-A^neo^ (expression of a dominant negative allele) and Total KO (MAO-A^−/−^)	**MAO-A deficiency in heart failure model was associated with compensated left ventricular function and hemodynamic stability** (1) Decreased the formation of hydrogen peroxide and ROS (2) Decreased level of fibrosis	(25–27)
MAO-B	Total KO (MAO-B^−/−^)	**MAO-B deficiency in heart failure model was associated with compensated left ventricular function and decreased levels of fibrosis and apoptosis** (1) Decreased levels of mitochondrial hydrogen peroxide (2) Maintained mitochondrial membrane potential	(26, 28)
SOD2	Total KO (apoE^−/−^/SOD2^+/−^)	**SOD2 deficiency was associated with increase in the number of atherosclerotic lesions** (1) Decrease SOD2 antioxidant activity (2) Increase mtDNA damage	(29)
SOD3	VSCM (SOD3^loxP/loxP^ × Tg^cr/SMMHC^)	**No effect of vasculature SOD3 on hypertension caused by Ang-II** (1) Increase vascular ${\text{O}}_{2}^{-\cdot }$ production (2) No further increase in ${\text{O}}_{2}^{-\cdot }$ production by Ang-II infusion (3) Modest reduction in endothelium-dependent vasorelaxation in Ang-II treatment (4) Marked reduction in NO bioavailability No further reduction in NO bioavailability by Ang-II infusion (5) No effect on basal blood pressure to Ang-II; in SOD3 VSMC deletion (6) Hypertensive response to Ang-II; in SOD3 CNS deletion	(30)
	Total KO (SOD3^−/−^)	**SOD3 plays a critical role in regulating O2**^−·^ **production** (1) Reduction in endothelium-dependent Ach relaxation (2) Increase O2^−·^ production	(31)
Atox1	Total KO (Atox1^−/−^)	**Atox1 is required for the full activation of SOD3 in a copper-dependent manner** (1) Using a cultured fibroblast, the activity of SOD3 was dramatically decreased (2) Using a cultured fibroblast, SOD3 mRNA was decreased (3) No effect on SOD3 protein levels or activity in a cultured fibroblast (4) Decrease in SOD3 activity and protein expression in the aorta	(32)
	Total and tissue specific KO (Atox1^−/−^ Total KO and EC-specific KO)	**The critical role of Atox1 in neovascularization and tissue repair** (1) Reduction in wound closure rate (2) Rapid epithelization (3) Decrease VEGF expression (4) Increase in levels of Cu content, reflecting delayed wound healing (5) Reduction in capillary count and blood flow (6) Reduction Mac3 macrophage with associated reduction in SDF1 alpha and VCAM1 (7) Loss of Atax1 in endothelium in endothelium-specific. (8) Increase in nuclear Atox1 [Atox1^−/−^ mice treated with gene transfer nuclear-targeted Atox1] (9) Reduction in p47phox expression (10) Reduction in ${\text{O}}_{2}^{-\cdot }$ production (11) Reduction in NF-kB activity (12) Reduction in cyclin D1 cells (13) Reduction in ECM accumulation	(33)
	Total KO (Atox1^−/−^)	**Critical role of Atox1 in regulating hypertension and vascular responses induced by Ang-II** (1) Infusion of Ang-II increase Atox1 protein expression, specifically in the nucleus (2) Inhibition of the increase in mRNA, protein, and activity of SOD3, but no effect on SOD1 (3) Increase in O_2_^−·^ production and hence the blood pressure after Ang-II infusion (4) Endothelial-dependent Ach relaxation and inhibiting Ang-II-induced vasoconstriction (5) Ang-II enhanced Atox1 translocation into the nucleus and the binding to SOD3 promoter (6) Translocation of Atox1-ATP7A-SOD3 complex to the plasma membrane (7) Decrease copper levels without the effect of Ang-II	(34)
ATP7A	Total KO (ATP7A^mut^)	**Decreased level of ATP7A contributes to endothelial dysfunction** Using type 1 diabetic mice: (1) Decrease in SOD3 activity, yet with increase SOD3 protein levels (2) Unaltered SOD1 activity and protein levels (3) Increase ${\text{O}}_{2}^{-\cdot }$ production (4) Restoration of SOD3 activity after copper addition (5) Reduction in endothelium-dependent Ach relaxation (6) Decrease expression of ATP7A (7) Insulin increase ATP7A expression and restore SOD3 activity	(31)
GPx-1	Total KO (GPx-1^+/−^) and (GPx-1^−/−^/ApoE^−/−^)	**Negative effects of GPx-1 deficiency** (1) Paradoxical mesenteric vasoconstriction (2) Decrease accumulation of cGMP within the aorta	(35)
		**Accelerated atherosclerotic lesion formation** (1)Increase oxidative stress within the vessel wall (2)Decrease NO bioavailability, and increased protein nitration	(36)
Trx2	Cardiac myocyte-specific KO (Trx2-cKO)	**Lack of txr2 cause the development of dilated cardiomyopathy and heart failure** (1) Increase in mitochondrial ROS production (2) Activation of ASK-1 and apoptosis of the myocardium Increase in heart size, reduced ventricular wall thickness with reduced contractility function	(37)

**Table 2:** Transgenic oxidant and antioxidant animals and their effects on cardiovascular system.

**Effector**	**Tissue/cell (model)**	**Effects and phenotypes**	**References**
NOX1	VSMC (Tg^SMCnox1^)	**The critical role during neointimal formation after vascular injury** (1) Enhance cell proliferation and migration (2) Augmentation of fibronectin production	(6)
		**Impaired vascular relaxation in response to NOX1 overexpression with Ang-II infusion** (1) Elevation of NADPH oxidase activity (2) Significant elevation of NADPH oxidase activity upon Ang-II infusion (3) Increase in blood pressure (4) Impairment of endothelium-dependent relaxation with Acetylcholine treatment, and exacerbated upon Ang-II infusion (5) Reduction in the amount of bioavailable NO levels, and exacerbated upon Ang-II infusion (6) Uncoupling of eNOS with reduction in the reduced form of BH_4_, and greater reduction upon Ang-II infusion; oxidized form showed a reciprocal change (7) BH_4_ has an antioxidant role by reducing the uncoupling of eNOS and improving endothelium-dependent relaxation in an Ang-II treatment	(38)
NOX2	Endothelium (Endothelial-Targeted Nox2 Transgene) binary (Tet-ON/OFF) conditional transgenic mouse (Tet-Nox2:VE-Cad-tTA) that induces endothelial cell (EC)-specific overexpression of Nox2	**Potentiation of the pressor response upon Ang-II infusion** (1) Increase in endothelial specific Tie2-NOX2 mRNA and protein (2) Increase expression of p22^phox^ mRNA and protein. (3) Increase ${\text{O}}_{2}^{-\cdot }$ production (4) Increase MnSOD protein (5) Increase eNOS protein (6) Phosphorylation of ERK1/2 (7) Increase systolic blood pressure upon acute and chronic Ang-II infusion	(39)
		**NOX2 overexpression increased NADPH oxidase activity leading to:** (1) ROS-mediated activation of AMPK-eNOS axis, which is CaMKK beta-dependent (2) Increased coronary microvessel dilation, which is AMPK-eNOS-mediated (3) Inhibition of mTOR signaling and activation of the protective Autophagy process	(3)
NOX4	Endothelium (Endothelial specific Nox4 mouse)	**Regulation of ischemic induced angiogenesis related signaling pathways** (1) Elevation of NOX4 expression in the endothelium (2) Increase in H_2_O_2_ and TGFbeta1 (3) Activation of eNOS, VEGF2R, p38MAPK, AMPK alpha pathways (4) Improvement of angiogenesis by regulating proliferation, migration, and network formation (5) Improvement of angiogenesis by TGFbeta1 independent of H_2_O_2_ (6) Contribution to VEGFA angiogenesis (7) Improvement of angiogenesis and endothelial cells survival in hypoxic conditions	(40)
		**NOX4-incduced H**_2_**O**_2_ **play a critical role in the determining the level of active TGF beta1** (1) PEG-catalase scavenger for NOX4-induced H_2_O_2_ in non-Tg (2) Increase in TGF beta1 precursor and decrease in the active TGF beta1	
		**TGF beta1- induced proliferation and migration independent of H**_2_**O**_2_ (1) Suppression of cell proliferation and migration with PEG-catalase treatment (2) No effect on the level of TGF beta1	
SOD1	Total Tg (SOD1-Tg)	**Cardiomyocyte survival and resistance to ischemic-reperfusion injury** (1) Reduction in ${\text{O}}_{2}^{-\cdot }$ production (2) Suppression of apoptosis (3) Reduction in sapsase-9 and caspase-3 activity (4) Reduction in FasL expression (5)Low levels of pro-inflammatory cytokines (6) Low serum level of CKP-MB (7) Less intimal thickening and preserved vessel lumen (8) Less MCP1/CCL2 and adhesion molecule	(41)
		**Protection against postischemic injury** (1) Increase total SOD1 expression and activity (2) Reduction in radicals (3) Higher contractile function recovery (4) Decrease cellular injury (5) Improvement in cardiac energetic state and metabolic recovery with less myocardial infarction (6) Sustained reperfusion time	(42)
Atox1	Total Tg (Atox1^+/+^)	**The critical role in regulating vascular SOD3** (1) Using a cultured fibroblast, the activity of SOD3 was increased (2) Using a cultured fibroblast, SOD3 mRNA was increased (3) No effect on SOD3 protein levels or activity in a cultured the fibroblast (4) Increase in SOD3 activity and protein expression in the aorta	(32)
Trx1	Cardiac-specific (Tg-DN-Trx1)	**The role of Trx1 in preventing cardiac hypertrophy** (1) Dominant negative (ND) mutant (C32S, C35S) of Trx1 diminished the activity of Trx1 (2) Elevation of oxidative stress (3) Cardiac hypertrophy Enhance Ras-Raf-1-ERK pathway	(43)
ATP7A	Total Tg (ATP7A-over-expressing transgenic mice)	**Protective role of ATP7A in endothelial dysfunction** (1) Increase on SOD3 specific activity Decrease in ${\text{O}}_{2}^{-\cdot }$ production (2) Improvement in Ach-induced endothelium-dependent relaxation	(31)

## Oxidants

There is compelling evidence that indicates involvement of ROS molecules in CVD and their progression (5). In the heart and vasculature, ROS are synthesized from different locations and enzymes including NADPH oxidases, XO, cytochrome P450 monooxygenase, monoamine oxidase (MAO) and from the uncoupling of NOS. Under normal conditions, some of these enzymes such as NADPH oxidase produce physiologic amounts of ROS that are involved in multiple signaling pathways (3, 5, 14). While other enzymes such as MAO generate ROS as byproducts during metabolism of other molecules (25, 28, 44–46). In disease conditions, the activity of most of these enzymes is upregulated, generating increasing amounts of ROS that are thought to be involved in the pathogenesis and progression of the underlying conditions. In this section, we will be addressing some of the transgenic and KO animal models that were used to demonstrate the roles and activities of the major ROS-generating enzymes in CVD.

### NADPH oxidase

NADPH oxidases have different isoforms, among them, NOX1, NOX2 (gp91^phox^), NOX4, and NOX5 are the major contributor in the vasculature (5). NADPH oxidase is a complex containing membrane bound and cytosolic subunits. The membrane bound subunits are NOX2 (gp91^phox^) and p22^phox^. The cytoplasmic subunits are p47^phox^, p67^phox^, and Rac1-containing GTPase (5). This multi-subunit enzyme complex is triggered by different stimuli including stress, hypoxia, hormones, or cytokines (47). Activation or inhibition of one or more of the regulatory subunits modulates ROS levels in CVS which in turn induces changes in sub-cellular signal transduction and determines CVD phenotype.

#### NADPH oxidase 1

NADPH Oxidase 1 (NOX1) is an enzyme expressed in CVS (9, 48–50). Several researchers have investigated specific role of NOX1 and the extent to which NOX1 contributes to cardiovascular pathophysiology. For example, NOX1 has been recently shown to be involved in Angiotensin-II (Ang-II) induced vascular responses and in CVD (7, 8, 51) (Tables 1, 2).

##### Ang-II induced-vascular responses

Ang-II plays a role in activating NADPH oxidases enzymes, especially NOX1 and NOX2, resulting in increase in ROS level (8, 38, 51). Ang-II is a critical component of renin-angiotensin system, which plays an important role in renal and cardiovascular pathophysiology (52). In CVS, Ang-II is involved in several functions such as: (i) potent vasoconstriction (ii) elevation of blood pressure (iii) increase in vascular smooth muscle cell (VSMC) proliferation and hyperplasia in the aorta (iv) participation in the process of oxidants formation resulting in vascular damage, and accumulation of extracellular matrix metalloproteinases (MMP), and (v) aortic aneurysm and dissection (8, 52–55).

Using different models of transgenic and knockout animals, NOX1 has been shown to play critical roles in cardiovascular pathophysiology, especially in augmenting and maintaining Ang-II responses (Tables 1, 2). In Tg^SMCnox1^ mice treated with Ang-II, blood pressure was elevated. One possible mechanism is reduction in nitric oxide (NO) bioavailability due to increase quenching of NO by ROS, hence impaired endothelial-dependent vessel relaxation (Table 2) (38, 56). This notion was further supported by the findings where a decrease in systolic blood pressure was observed in NOX1^−/−^ mice treated with Ang-II (Table 1) (7). Taken together, NOX1 appears to have a critical role in modulating Ang-II-induced vascular response and hypertension. In NOX1^−/−^ mice, both aortic medial hypertrophy and extracellular matrix accumulation were attenuated, suggesting a crucial role of NOX1 in VSMC proliferation and MMP synthesis (7, 8).

##### Neointimal injury

VSMC is the main element in neointimal injury. The abnormal growth of neointimal tissue in the blood vessels contributes to the development of vascular occlusive diseases (6, 9). Following an injury or endothelial breach of continuity, VSMCs migrate to the damaged area and proliferate. Platelet-derived growth factor (PDGF) and reduction in NO have been believed to play critical roles in this process. ROS production from the injured tissue has also been considered a major player in neointimal growth (6). As mentioned previously, NOX1 in VSMC is one of the main sources of ROS (6, 9). In NOX^−/−^ mice, cell proliferation in the setting of an injury-induced neointimal formation was inhibited. Interestingly, whereas apoptosis, fibronectin accumulation and migration of VSMC were reduced in the KO model (6), cell proliferation, migration, and fibronectin production were augmented in NOX1 overexpressing Tg^SMCnox1^ mice (9). These findings suggest that NOX1 is essential for neointimal formation. Intriguingly, NOX1 seems to have a role in regulating matrix components as fibronectin production is reduced when NOX1 was knocked out, and augmented when it was expressed. Furthermore, NOX1 was shown to be involved in VSMC migration through coiflin. In NOX1^−/−^, coiflin was phosphorylated and Serine/threonine-protein kinase (PAK1) levels were reduced, explaining coiflin's involvement in the migration process (6).

##### Aortic dissection and aneurysm

Another important cardiovascular pathology is formation of aneurysm and aortic dissection. In order to understand its pathobiology, NOX1-deficient mice were treated with Ang-II and were shown to have reduction in aortic dissection and aneurysm (57). In this model, inhibition of MMP was observed (8) and the number of sudden death of mice was significantly reduced. Together, these findings suggested that NOX1 was involved in Ang-II induced aortic aneurysm and dissection.

##### Atherosclerosis

Atherosclerosis is a condition characterized by plaque formation in the medium and large blood vessels and is considered as one of the primary causes of CVD (58). In ApoE^−/−^ and NOX1^−/−^ animals, a reduction in the atherosclerotic area and the macrophages within the plaque were observed, suggesting a crucial for NOX1-induced ROS formation in the pathogenic process of ApoE-mediated atherosclerosis (6).

#### NADPH oxidase 2

NOX2 (gp91^phox^) is a major membrane-bound subunit of the NADPH oxidase enzyme. Like NOX4, NOX2 localization is not limited to the cellular membrane, it is found in different subcellular compartments such as the perinuclear membrane and the endoplasmic reticulum (ER) (5). NOX2 has been implicated in the pathogenesis of CVD including atherosclerosis (10, 11). However, recent studies have shown beneficial effects for the endothelial cell-specific overexpression of NOX2 on endothelial function and proliferation (3, 4).

Using NOX2 deficient (NOX2^−/−^) animal model, intimal thickening following arterial injury was found to be reduced by 34% compared to the wild-type (WT) counterpart (11). In this study, progressive thickening of the medial arterial layer was observed up to 28 days after the injury in both NOX2 deficient and wild-type mice. Whereas the intima/media ratio (I:M ratio) increased from 0.24 at day 7 to 0.97 at the 28th day from the injury in wild-type mice, the I:M ratio was 40% lower by day 28 in the NOX2 deficient mice compared to WT (11). These findings suggest an important role for NOX2-containing NADPH oxidase in inducing intimal thickening and cellular proliferation after vascular injury. Furthermore, altered leukocyte infiltration was observed in NOX2^−/−^ mice suggesting that NOX2 plays critical roles in the accumulation of inflammatory cells within the vessel wall in response to injury (11).

In another study, [(ApoE^−/−^)/(Nox2^y/−^)] double knockout mice were used to examine the effects of NOX2-containing NADPH oxidase on the development of atherosclerosis in an atherogenic, ApoE-deficient mice (10) (Table 1). Although NOX2 knockout in [(ApoE^−/−^)/(Nox2^y/−^)] mice had minimal effect on the plasma-lipid levels compared to (ApoE^−/−^) mice, the area of atherosclerotic lesions in the aorta was 50% less in [(ApoE^−/−^)/(Nox2^y/−^)] compared to (ApoE^−/−^) mice (10). The decrease in the atherosclerotic plaque area was associated with decreased ROS levels. NO bioavailability was improved, and superoxide level was reduced by 75% in [(ApoE^−/−^)/(Nox2^−/−^)] mice compared to (ApoE^−/−^) animals (10). These results suggest that NOX2-containing NADPH oxidase enzyme-induced ROS production and resulting effects on NO bioavailability within the vessel wall has significant effect on the atherosclerotic plaque size (10).

To examine the effect of NOX2-containing NADPH oxidase on the vascular oxidative stress and response to hemodynamic alterations, Tie2-NOX2 transgenic mouse model was developed (39) (Table 2). The transgenic mouse showed an overall increase in NADPH activity resulting in increased ${\text{O}}_{2}^{-\cdot }$ production (39). With the increased amount of ${\text{O}}_{2}^{-\cdot }$, there was a compensatory upregulation of Manganese-dependent Superoxide Dismutase (MnSOD) (SOD2) and eNOS protein followed by activation of the downstream signaling pathway ERK1/2 (39). Interestingly, the authors reported a lack of effect of NOX2-Tg on the basal blood pressure, however, it had greatly potentiated the pressor response upon acute and chronic Ang-II infusion (39). Together, these findings suggest an important role in modulation of the hemodynamic response to Ang-II by NOX2-containing NADPH oxidase (39).

Increased levels of ROS have been found to be associated with several cardiac diseases (3, 4). However, several clinical trials using global antioxidants have failed to improve and rather sometimes have worsen the outcome of CVD (3–5). In addition, decreased endothelial ROS level resulted in PI3K-Akt-eNOS inhibition and impairment of endothelium-dependent coronary vasodilation (3). These paradoxical findings suggest that ROS may also have positive effects on endothelial cells (EC). To examine the hypothesis, a novel binary (Tet-ON/OFF) conditional transgenic mouse (Tet-Nox2:*VE-Cad-tTA*), which induces endothelium-specific generation of Nox2-dependent NADPH oxidase-derived ROS was developed (3, 4). Isolated coronary microvessels from Tet-OFF mice showed significant increase in coronary vasodilation, upon stimulation by VEGF or Acetylcholine compared to Tet-ON vessels (5). The increased coronary vasodilation in Tet-OFF coronary microvessels was found to be the result of ROS-mediated activation of the AMPK-eNOS axis, which resulted in increased levels of NO in vascular endothelium. ROS-induced activation of AMPK showed inhibitory effect on mTOR signaling and subsequent activation of the protective autophagy process resulting in enhanced survival of Tet-OFF mouse heart endothelial cells (MHEC) (3).

To examine whether the duration of exposure to high levels of ROS dictates the ultimate effect on the vascular endothelium, Tet-ON and Tet-OFF animals were divided into short-term (8 weeks) and long-term (20 weeks) groups. Interestingly, the coronary microvessels isolated from animals that are 8 weeks Tet-OFF showed 22% increase in vasodilation, whereas those isolated from animals that were 20 weeks Tet-OFF showed 44% reduction in vasodilation in response to Acetylcholine and VEGF, compared to Tet-ON animals (4). MHEC from animals that were Tet-OFF for 20 weeks showed 2-fold increase in the level of peroxynitrite compared to the short-term exposure group (4). This finding indicates that long-term exposure to higher levels of ROS resulted in superoxide and NO interaction, leading to the formation of peroxynitrite and impairment of the ROS-activated AMPK-eNOS-mediated vasodilation. On the contrary, short-term exposure to ROS in Tet-OFF animals increased EC proliferation and endothelial sprouting from the aorta, while long-term exposure inhibited EC proliferation and aortic sprouting (4). The long-term exposure to high levels of ROS in Tet-OFF animals resulted in increased levels of mitochondrial ROS (mtROS). This led to mitochondrial damage by altering the mitochondrial membrane potential and significant reduction in the mitochondrial DNA (4). Increase in mtROS in short-term Tet-OFF animals was counteracted by the increased expression and activity of SOD2 enzyme. SOD2 was found to undergo nitration modification when exposed to long-term ROS increase, leading to the enzyme's inhibition (4). Together, these results demonstrated that duration of ROS exposure on EC is critical in determining phenotypic effects of ROS on blood vessels.

#### NADPH oxidase 4

NADPH Oxidase 4 (NOX4) enzyme is expressed in endothelial as well as vascular smooth muscle cell (59). NOX4 can generate ROS, specifically H_2_O_2_, without requiring activating cytosolic factors (5, 59). Several studies have implicated role of H_2_O_2_ in cardiovascular cell survival.

Animal models with NOX4 transgene have been shown to have higher levels of H_2_O_2_ resulting in enhanced activation of eNOS, VEGFR2, p38MAPK, and AMPK-alpha pathways (40, 60, 61). These signaling pathways are critical in inducing angiogenesis by regulating proliferation, migration, and tubular network formation by ECs (40, 60). NOX4^−/−^ mice demonstrated reduction in hemoxygenase-1 (HO-1) accompanied by an increase in apoptosis and increase in E-Cadherin expression (61). These finding suggested an important role for NOX4 in regulating HO enzyme and thus heme degradation.

### P47^phox^

P47^phox^ is one of the cytosolic subunits of the NADPH oxidase enzyme (17). p47^phox^ has been shown to have a pivotal role in the development or progression of several diseases, for example, atherosclerosis and hypertension (15, 16, 62).

Using knockdown model, researchers demonstrated that p47^phox^ is involved in cardiovascular pathology through multi-subunit NADPH oxidase enzyme complex (Table 1). In p47^phox−/−^ animals, level of NADPH oxidase enzyme-derived ROS production was inhibited (14, 17). This resulted in the reduction of PI3K-Akt-eNOS, NO production, and VEGF-induced vasodilatation (14). In ApoE^−/−^ and p47^phox−/−^ model, there was a significant decrease in atherosclerotic lesion (16, 62). Additionally, it was also shown to be involved in neointimal hyperplasia (16, 17). These findings suggested that p47^phox^ plays a crucial role in the development of atherosclerosis and progression of neointimal hyperplasia. Another study using KO model of p47^phox−/−^ mice showed that there was a reduction in ROS formation as well as reduction in Ang-II-mediated increase in blood pressure, suggesting a pivotal role for NADPH-p47^phox^ in mediating Ang-II-induced hypertension (15).

### Xanthine oxidoreductase

Purines catabolism is carried out by the enzyme XO, which degrades adenine to hypoxanthine and guanine to xanthine (63, 64). Hypoxanthine and xanthine are then oxidized by XO, catalyzing the oxidation of hypoxanthine to xanthine and subsequently to uric acid (65). Both steps require oxygen, forming the superoxide molecules, which are then converted to hydrogen peroxide (64, 65). In human, the inactive state of the enzyme made determination of the enzyme distribution difficult, yet it was identified in the liver, small intestine, and mammary glands. It has been identified in other mammalian tissues as well as in bovine and rat heart (66–68).

The byproducts (i.e., superoxide and hydrogen peroxide) of XO enzyme are believed to have detrimental effects on heart. XO has also been associated with endothelial dysfunction and was found to be elevated during ischemia-reperfusion (IR) injury (69). Using rat heart, researchers showed that there was an elevation of H_2_O_2_ levels and associated left ventricular dysfunction in IR injury (70). Rabbit heart with ischemia experienced less ventricular dysfunction when treated with allopurinol (XO inhibitor) compared to untreated heart (71). Additionally, in spontaneous hypertensive/heart failure rat model, chronic use of XO inhibitor induced reverse cardiac remodeling and preserved the structure and function of the heart (72), suggesting critical roles of XO in IR and cardiac remodeling. Several clinical trials are being conducted to determine the effect of XO inhibitor in patients with CVD (73–75). Precise mechanisms by which XO exerts its effects on the CVS and how XO inhibitor can attenuate the adverse effects remain yet to be elucidated.

### Nitric oxide synthase

NO is one of the major effector molecules in cardiovascular physiology (76). In addition to its critical role in inducing endothelium-dependent vasorelaxation, it also inhibits platelet aggregation, vascular smooth muscle proliferation, and leukocyte adhesion (18, 76, 77). It is important to maintain an adequate level of NO for the integrity of the vasculature and prevention of atherosclerosis (18, 76). Hypertension, atherosclerosis and other CVD have been linked to altered NO bioavailability and abnormalities in NO signaling (76).

There are three isoforms of NOS which produce NO: endothelial NOS (eNOS), Inducible NOS (iNOS) and Neuronal NOS (nNOS). These enzymes are encoded by distinct genes, yet they share similar mechanisms and sites for cofactor binding (76). They all require transfer of electron from oxygen to catalyze the conversion of L-arginine to L-citrulline (76). Under normal conditions, eNOS is the major isoform expressed within the vasculature. However, all three isoforms have been found to be overexpressed in atherosclerotic lesions (19, 24, 78). Previous studies have shown that nNOS and eNOS have protective roles against artificially induced and spontaneous atherosclerosis (18, 19, 23, 24), while iNOS expression aggravated progression of atherosclerotic plaque (20).

#### Endothelial nitric oxide synthase

eNOS enzyme is thought to have a protective role against the development of atherosclerotic lesions. Although eNOS has beneficial effects on CVS, uncoupling of eNOS results in ROS production instead of NO synthesis. Different studies have investigated the effects of eNOS deficiency in the context of atherosclerosis, using the atherogenic (ApoE^−/−^) mice as a model (19, 77). In one study, expression of VCAM-1 by endothelial and smooth muscle cells was reported to be increased in [(ApoE^−/−^)/(eNOS^−/−^)] compared to (ApoE^−/−^) mice, resulting in increased Leukocyte-Endothelial interaction. The mononuclear accumulation within the vessel wall was also increased in this animal (19). Interestingly, genetic deletion of eNOS in ApoE deficient mice resulted in significant reduction in superoxide level compared to wild-type mice, suggesting that eNOS contributed to the formation of superoxide in the atherogenic (ApoE^−/−^) mice (19). Additionally, inhibition of eNOS activity resulted in a significant reduction in superoxide level in (ApoE^−/−^) but not in wild-type or [(ApoE^−/−^)/(eNOS^−/−^)] mice, further confirming that the increased level of superoxide in (ApoE^−/−^) mice was the result of eNOS uncoupling (19).

#### Inducible nitric oxide synthase

iNOS expression in the atherosclerotic lesion is limited to the mononuclear leukocytes and smooth muscle cells (20). Whereas the activities of eNOS and nNOS are regulated by the intracellular calcium concentration, iNOS activity is mostly regulated by cytokines and bacterial endotoxins released under pathological conditions. Using the hyperlipidemic ApoE deficient mice model, several studies have investigated the role of iNOS in the atherosclerotic plaque formation (20, 21, 23).

Although body weight and total cholesterol and triglyceride levels were not different between ApoE-KO and ApoE/iNOS-dKO mice fed on high fat diet, the total area of the atherosclerotic lesion was significantly decreased in ApoE/iNOS-dKO mice compared to their ApoE^−/−^ controls (20, 22), suggesting a deleterious role for iNOS in CVD. The reduction in the area of atherosclerosis was found to increase over time, suggesting that protection from lesion formation increases over time in iNOS deficient mice (20). The decrease in atherosclerotic lesion formation seen in ApoE/iNOS-dKO mice was accompanied by decreased plasma levels of lipoperoxide, suggesting that iNOS-mediated oxidative stress was also decreased in this animal (20). Oxidized low density lipoprotein (oxLDL) was found to induce accumulation of the macrophage-derived foam cells within the atherosclerotic plaque by increasing iNOS-mediated oxidative stress (21). Treatment with oxLDL resulted in increased iNOS expression, peroxynitrite formation and significant decrease in cell migration, which was reversed upon co-administration of iNOS inhibitor (21), further supporting a role for iNOS in atherosclerosis.

#### Neuronal nitric oxide synthase

In early and advanced atherosclerotic lesion, nNOS is expressed in endothelial cells, macrophages and smooth muscle cells (23). Several studies have demonstrated a protective role for nNOS in atherosclerosis (23, 24). Using a mouse model, in which unilateral carotid artery ligation was performed to induce vascular injury, it was shown that neointimal formation and vasoconstriction were significantly accelerated in nNOS-KO mice compared to their wild-type littermates (24). These results suggested a protective role for nNOS against the development of atherosclerosis.

The nNOS gene produces multiple mRNA splice variants, leading to the formation of four distinct proteins (24). In order to study the role and activity of nNOS in spontaneous atherosclerosis, nNOS deficient mice model (nNOS-KO) was combined with the atherogenic ApoE-KO mice model (23). Total lesion area was significantly increased in male ApoE/nNOS-dKO mice compared to their ApoE-KO controls at 14 weeks of western-type diet, while there was no difference in the lesion area of the female animals (23). However, the lesion area of female ApoE/nNOS-dKO mice was markedly increased after 24 weeks of western-type diet compared to ApoE-KO female mice, plausibly due to the reduced blood pressure observed in ApoE/nNOS-dKO female mice (23). These results suggested a vasculo-protective role for nNOS in atherosclerosis.

### Monoamine oxidase

MAO are enzymes located on the outer membrane of the mitochondria. These enzymes are responsible for the metabolism of catecholamines including epinephrine and norepinephrine, and other biogenic amines such as serotonin (25, 28, 44, 79). There are two types of MAOs, MAO-A, and MAO-B, which are different in their substrate and inhibitor sensitivity (25, 26, 28, 44, 79). MAO-A is the predominant isoform expressed in the cardiac tissue of humans and rats, while MAO-B is also abundant in the myocardium of mice and humans (26, 28, 79, 80). Under stressful conditions, such as in congestive heart failure, the sympathetic tone and circulating catecholamines are increased leading to upregulation of MAO activity due to the increased substrate availability (25, 28, 79). Metabolism of catecholamines and other biogenic amines by MAO enzymes results in the formation of hydrogen peroxide, aldehyde intermediates and ammonia as end products (28, 79). Due to their ability to generate increasing levels of ROS, MAOs have been considered as the major contributors to the oxidative damage in several CVD such as heart failure and IR injury (25, 28, 44–46).

#### Monoamine oxidase A

Being a major source of hydrogen peroxide and ROS under stressful conditions, both genetic deficiency and pharmacological modulation of the enzymatic activity of MAO-A have been found to improve the outcome of different pathological conditions (25, 44). A study showed that treatment of rat cardiomyocytes with serotonin resulted in mitochondrial dysfunction and apoptosis (44). These deleterious effects were fully prevented upon treating the cells with Pargyline, a MAO inhibitor. This suggests that the serotonin-induced apoptosis and mitochondrial dysfunction are mediated by the accumulation of hydrogen peroxide generated by MAO-A. Moreover, the infarction size following an ischemic injury was significantly reduced in rats treated with MAO inhibitors compared to those treated with vehicle only (44).

Another study demonstrated that treatment of isolated cardiomyocytes with Norepinephrine (NE) resulted in increased MAO-A expression, ROS production and cellular hypertrophy (25). The concomitant administration of MAO inhibitor prevented NE-induced ROS production and cellular hypertrophy. Furthermore, the administration of MAO inhibitor to a heart failure mouse model enhanced preservation of the left ventricular function and decreased heart weight (25). The genetic deficiency of MAO-A due to the expression of a dominant negative allele (MAO-A^neo^) was found to be associated with improved ventricular function and decreased level of fibrosis (25, 26). Similarly, (MAO-A^−/−^) mice were found to have compensated left ventricular function and hemodynamic stability despite the presence of hypertrophic myocardium and ventricular dilation (26, 27). These results suggest that MAO-A-mediated oxidative stress contributes to the impairment of the cardiac function in heart failure.

#### Monoamine oxidase B

Until recently, most studies focused on addressing the role of MAO-A in heart diseases because it was believed to be the predominant isoform expressed in the cardiac tissue of humans and rats (26, 28, 79, 80). As stated earlier, MAO-B is also abundantly present in the human heart, and there are several labs currently studying MAO-B to elucidate the role of MAO-B in CVD. To that end, using MAO-B deficient mice (MAO-B^−/−^), trans-aortic constriction was performed to induce heart failure. Whereas both (MAO-B^−/−^) and wild-Type (WT) mice were found to have ventricular hypertrophy following the trans-aortic constriction, only (MAO-B^−/−^) mice maintained compensated left ventricular function (28). Additionally, level of dopamine was greatly reduced in the heart tissue of WT mice in spite of the unchanged MAO-B protein expression (26, 28). Together, these findings suggested that activity of the enzyme was upregulated upon exposure to higher levels of the substrate. Fibrosis and apoptosis were markedly increased in WT mice compared to their (MAO-B^−/−^) counterparts (26, 28). Cardiomyocytes of WT mice with dopamine showed significant increase in mitochondrial hydrogen peroxide levels and significant drop in the mitochondrial membrane potential. These effects were ameliorated upon co-incubation with a MAO-B inhibitor and were completely absent in MAO-B deficient mice (28). Together, these findings suggested that MAO-B plays a critical role in inducing mitochondrial oxidative stress and dysfunction in CVD.

## Antioxidants

Antioxidants include both enzymatic and non-enzymatic antioxidants that work as a line of defense in protecting the tissues from the oxidative damages. Thus, antioxidants are critical for the maintenance of homeostatic balance of oxidants at the tissue levels. When levels of ROS exceed physiological limit and the antioxidants become overwhelmed, the resulting impaired balance affects cellular functions (81, 82). There are several important intracellular enzymatic antioxidants that include SODs, glutathione peroxidase, and thioredoxin (82). To conceptualize the importance of antioxidants and equivalent molecules in CVD, transgenic and knockout models have been utilized to elucidate the functional roles of each of these proteins.

### Superoxide dismutase

SODs are copper-containing and zinc-containing enzymes (32). They convert ${\text{O}}_{2}^{-\cdot }$ to H_2_O_2_, minimizing the damaging effects of superoxide. There are three isoforms: (i) cytoplasmic CuZnSOD (SOD1) (ii) mitochondrial MnSOD (SOD2), and (iii) extracellular SOD (SOD3) (40).

#### Superoxide dismutase 1

Superoxide dismutase 1 (SOD1) is a cytoplasmic zinc- and copper-containing enzyme that catalyzes the conversion of ${\text{O}}_{2}^{-\cdot }$ to H_2_O_2_ intracellularly (83). Subsequently, H_2_O_2_ is reduced to water and molecular oxygen by glutathione peroxidase or catalase (42, 84). Several studies have examined the role of SOD1 in IR injury and whether the overexpression of SOD1 is detrimental or beneficial to cells under the pathophysiological conditions with higher oxidative stress (42, 84, 85).

##### Ischemia-reperfusion injury

IR injury is one of the major causes of pathologies seen in the heart. It has been also linked as one of the prime complications of peri-transplant injury during heart transplantation (41). Ischemia denotes to the sudden cessation of blood supply to the organ, resulting in cellular damage (86). SOD1 was shown to protect from IR injury (41, 42, 84). In animal models overexpressing SOD1, the levels of ROS, specifically ${\text{O}}_{2}^{-\cdot }$, generated in post-ischemic tissues were significantly diminished compared to WT animals (42, 84). One of these models further showed improvement in the myocardial energetic state, metabolic recovery and reduction in myocardial infarction size (42). In another model with SOD1 overexpression, inflammatory cytokines, adhesion molecules, apoptotic proteins including caspase-9, caspase-3, and FasL were all reduced (41, 84). These results implicated SOD1 in reducing cardiomyocyte apoptosis and inflammation (42, 84). Taken together, all of the above findings suggest a critical role for SOD1 in minimizing IR injury in post-ischemia and post-transplantation settings (41, 42, 84). Other studies have thoroughly investigated the effects of exogenous administration of SOD1 on ischemic tissues, which was associated with dose-dependent toxic effect. This necessitates further studies aimed at examining whether SOD1 protect or potentiate adverse cellular injury in CVD (87).

#### Superoxide dismutase 2

SOD2 constitutes the major mitochondrial antioxidant defense system against superoxide radicals produced by the leakage of electron transport chain (88). Like other SOD enzymes, SOD2 catalyzes the conversion of superoxide to hydrogen peroxide. Deficiency in SOD2 is associated with oxidative mitochondrial damage, which is mediated primarily by superoxide. Whereas homozygous SOD2 deficiency (SOD2^−/−^) is not compatible with life and produces lethal mice phenotypes (88), heterozygous deficiency (SOD2^−/+^) has been associated with increased mitochondrial damage in the heart (89).

Mitochondrial DNA (mtDNA) damage is an indicator of ROS-mediated cellular damage (29). A study has addressed the question whether mtDNA damage plays a causal role in atherosclerosis or atherosclerosis results from the oxidative damage happening at the site of the lesion. Researchers used single KO (ApoE^−/−^) and double KO [(ApoE^−/−^)/(SOD2^+/−^)] mice models for this study (29). Results showed that aortic tissue from (ApoE^−/−^) mice had significant increase in mtDNA damage during the earliest stages of the atherosclerotic lesion development (29). [(ApoE^−/−^)/(SOD2^−/+^)] mice showed significant increase in the number of atherosclerotic lesions compared to their (ApoE^−/−^) counterparts (29). Interestingly, [(ApoE^+/+^)/(SOD2^+/−^)] mice did not develop atherosclerotic lesions at all, suggesting that the phenotype observed in [(ApoE^−/−^)/(SOD2^+/−^)] mice resulted from the combined effect of having an atherogenic environment and increased mitochondrial ROS levels (29).

#### Superoxide dismutase 3

Superoxide Dismutase 3 (SOD3) is a major extracellular antioxidant enzyme. It is synthesized inside the cell and anchored in the extracellular matrix and also on endothelial surface (31, 90–93). This enzyme acts as a first line of defense against ROS because of its extracellular location. Similar to other SODs, it catalyzes the conversion of ${\text{O}}_{2}^{-\cdot }$ to H_2_O_2_. The regulation of redox balance highlights the critical role of SOD3 in the pathogenesis of CVD.

SOD3 requires a catalytic copper for its full activity and since the intracellular copper levels are restricted, a soluble copper carrier is required to deliver the copper to this extracellular enzyme. This is achieved by the antioxidant-1 copper chaperone (Atox1) that interacts with ATP7A. Atox1-ATP7A pathway helps in the activation of the secretory pathway for copper as well as regulates the level of intracellular copper by excreting excess amount (83, 94, 95). Thus, ATP7A delivers copper to SOD3 to scavenge ${\text{O}}_{2}^{-\cdot }$ and increase bioavailability of nitric oxide to preserve the endothelial functions (31).

In SOD3^−/−^ mice, increase in ROS levels were reported [80.87] (Table 1). Increase in ROS was also observed in Atox1^−/−^ and ATP7A^mut^ mice, further supporting the physiological link of Atox1- ATP7A-SOD3 (30, 31, 83). In the knockdown animal model of SOD3 or ATP7A, a reduction in endothelium-dependent acetylcholine-mediated vasorelaxation, as a consequence of increased ROS production, was reported (31). SOD3^loxP/loxP^ × Tg^cr/SMMHC^ animals treated with Ang-II showed a marked reduction in nitric oxide bioavailability (30, 96). However, in a VSMC-specific SOD3 KO model, there was no effect of lack of SOD activity on Ang-II-induced hypertension (96), suggesting SOD3 plays a major role specifically in endothelial NO homeostasis by modulating ROS levels.

Since Atox1 plays a role in the regulation of SOD3, Atox1^−/−^ mice were used to see the endothelial cell specific role during wound healing. Atox1^−/−^ mice showed delay in wound healing which could be explained by the loss of copper sensor function implicated by increase copper content. These animals also showed a reduction in the number of capillaries, blood flow, Mac3+ macrophage, stromal cell-derived factor 1 (SDF1) alpha, and vascular cell adhesion molecule 1 (VCAM-1). These mediators are critical for neovascularization and tissue repair (34). Interestingly, Atox1^−/−^ demonstrated reduction in p47^phox^ expression, NF-kB activity and cyclin D levels. Together, these findings suggested a role for Atox1 in the downstream regulation of NF-kB via p47^phox^, and in cell proliferation via cyclin D, resulting in delay in wound healing.

### Glutathione peroxidase

Glutathione and GPx enzymes are amongst the most important antioxidant systems in mammalian cells (36). They play crucial roles in the defense against oxidative stress. The most abundant cellular form of GPx is GPx-1 [591]. GPx-1 converts hydrogen peroxide to water and molecular oxygen, and converts lipid peroxides to their respective alcohols using glutathione as a reducing agent (33, 36). Lipid peroxides can react with NO forming lipid peroxynitrite, which results in decreased NO bioavailability (33). Deficiency in GPx-1 has been linked to the development of endothelial dysfunction, inflammation and neointimal formation, suggesting a quenching role of ROS against NO (33, 35). GPx-1 deficiency was found to have deleterious effects also in the context of atherosclerosis (36).

GPx-1 and glutathione have been shown to have vasodilatory effects on the vasculature (33). Studies showed that infusion of wild-type mice with bradykinin or b-methacholine lead to dose-dependent vasodilation of the mesenteric arterioles *in vivo*. On the contrary, superfusion of the GPx-1 deficient mice with the same vasodilators resulted in paradoxical vasoconstriction of the mesenteric arterioles (33). Interestingly, superfusion of both animal types with the NO donor, sodium nitroprusside (SNP), resulted in dose-dependent vasodilation, suggesting that the lack of GPx-1 was associated with decreased NO bioavailability (33). Moreover, upon stimulation with Bradykinin, aortic tissue from GPx-1 deficient mice showed decreased accumulation of cGMP compared to wild-type aortic tissue, suggesting Bradykinin's involvement in the activation of NO-cGMP signaling cascade (33).

The double knockout [(ApoE^−/−^)/(GPx-1^−/−^)] mice developed more aggressive atherosclerosis compared to their (ApoE^−/−^) controls (36). There was marked increase in ROS levels within the vessel wall, mostly in the endothelium of the double-knockout animals compared to the controls (36). A decrease in NO bioavailability was also associated with increased protein nitration within the atherosclerotic lesions of [(ApoE^−/−^)/(GPx-1^−/−^)] compared to (ApoE^−/−^) mice (36). These findings suggest that GPx-1 deficiency is associated with accelerated atherosclerotic lesion formation and extensive oxidative stress within the plaque formation (36).

### Thioredoxin

Thioredoxin (Trx) is a small ubiquitin enzyme that serves as a reducing agent. Trx has been shown to be efficient in keeping a reduced state intracellularly (97–99). Trx maintains cellular functions via different mechanisms; primarily, it operates by reducing ROS and thereby protecting the cells from deleterious effects. It also plays a role in regulating apoptosis and inflammatory response (100, 101). Trx is active in the reduced form and operates by scavenging ROS and inhibiting apoptosis through inhibitory binding to apoptosis signal-regulating kinase-1 (ASK-1) (98, 100). Trx enzyme requires Thioredoxin Reductase (TrxR) to regenerate the reduced form of Trx at the expense of NADPH molecule (102). There are at least three isoforms of Trx in mammals: cytosolic/nuclear thioredoxin 1 (Trx1), mitochondrial thioredoxin 2 (Trx2), and sperm thioredoxin (SpTrx) (100–102). In the following sections, we will only discuss Trx1 and Trx2, and their roles in cardiac hypertrophy, IR injury, and heart failure.

#### Thioredoxin-1

Human Trx1 is a ubiquitous cytosolic protein, which can translocate to the nucleus or exit from the cell to the extracellular environment (103). It harbors two cysteines in the catalytic site (Cys-32 and Cys-35) and three additional non-catalytic cysteines (Cys-62, Cys-69, and Cys-73) (100). Under oxidative conditions, the cysteines in the catalytic site (Cys-32 and Cys-35) react with the oxidized substrate, generating a reduced substrate and an oxidized Trx1. The non-catalytic cysteines may suppress the enzymatic activity of Trx1 as Cys-62 and Cys-69 attenuate accessibility of TrxR to Trx1 by forming a disulfide bond, and Cys-73 can react with glutathione to form a mixed disulfide moiety (glutathionylation) (104, 105). Additionally, Cys-69 may also preserve the redox activity of Trx1 via nitrosylation (106).

##### Cardiac remodeling

The term “Remodeling” was first used to describe the replacement of the infarcted tissue with scars in myocardial infarction. Nowadays, the term is used more broadly to include adaptive and pathological changes of the heart after an injury (107). ROS have been shown to influence the progression of this process from the development of hypertrophy to compensated hypertrophy and subsequently to heart failure (107). Since ROS can induce hypertrophy, antioxidants such as Trx1 should logically suppress cardiac hypertrophy. In transgenic mouse overexpressing Trx1, myocyte hypertrophy was suppressed by Trx1-mediated inhibition of Ras-MAPK signaling pathway (43). Using adult rat ventricular myocyte, Trx1 was shown to inhibit activation of Ras by reducing thiol in Ras (108).

Another phenomenon that occurs in response to cardiac ischemia is the ischemic preconditioning. It has been described as a transient ischemia inducing a powerful protective mechanism against the longstanding ischemic insult in IR injury (109). It has been reported that ischemic preconditioning requires ROS production, which in turn induces protein kinase C (100, 110). Using *ex vivo* rat heart that overexpresses Trx1, it was demonstrated that Trx1 is essential for the cardiac ischemic preconditioning (111).

Prolonged ischemic injury increases ROS levels, resulting in the imbalance between the levels of ROS and NO, and consequently inactivation of Trx1 through post-translational modifications (106). Increased levels of ROS induce both apoptosis and fibrosis of cardiomyocytes (112, 113). As mentioned earlier, Trx1 exerts anti-apoptotic effect through ASK-1 and Akt signaling pathways (98, 100, 114). Trx1 activates Akt which in turn inhibits Bad (pro-apoptotic protein) and procaspase-9 (115). Together, Trx1 prevents pathological cardiac remodeling through intracellular signaling molecules that inhibits apoptosis and cell death. Further experiments are ongoing in several labs to elucidate the precise molecular mechanisms by which Trx1 modulate pathophysiology of CVD.

#### Thioredoxin-2

Trx2 is a mitochondrial protein, hence the name mitochondrial thioredoxin (100, 102). Like Trx1, it contains an active catalytic site but lacks the other non-catalytic cysteines (100). Due to high demand of ATP generation and oxygen consumption in the heart, mitochondria become susceptible to oxidative stress during highly active oxidative phosphorylation. Trx2 plays a critical role in protecting the mitochondria by respiration-dependent H_2_O_2_ removal in the organelle (116, 117). Mice with cardiac-specific ablation of Trx2 developed fatal cardiomyopathy (37). This was correlated with increased ASK-1 signaling and apoptosis of cardiomyocyte. These findings suggested that Trx2 helps preserve and maintain the integrity of the mitochondria in the heart with increased ROS levels (37, 118).

## Limitations

The availability of animal models has undeniably advanced our current knowledge and offered novel insights into different aspects of CVD and interventions. Yet there are certain limitations of the studies that utilize animal models. A major limitation for using small animals like mouse is lack of the translational aspects of these studies and lack of reliability of the outcomes of such studies for clinical applications and interventions (119, 120). Additionally, the size of the animal restricts the amount of blood sampling and complicates vessel dissection (120). Other concerns include different etiology of diseases among different species, and sometimes the number of samples in a study can be limited due to imposed financial constraints (121, 122). Thereby, it is imperative to follow a rational choice of animal models, and to carefully interpret the outcomes of a study. Moreover, there exist some enzyme-specific limitations. In this review, any enzyme-related limitation has been mentioned under each corresponding enzyme to aid the understanding and the articulation of thoughts for the readership.

## Concluding remarks

CVD are the leading cause death in the world (3). Increased levels of ROS have been associated with the development and progression of CVD. Several groups of researchers hypothesized that halting ROS production and augmenting antioxidant levels can have a promising outcome in the treatment of CVD (3). On the contrary, the findings of more recent studies do not support this notion. In this review article, we have presented the studies that have taken objective approaches to examine these opposing hypotheses by utilizing transgenic and knockout models of oxidant producing and antioxidant enzymes and their effects on cardiovascular health and disease.

This review has systematically cataloged the phenotypic outcomes in transgenic and knockout models of the same or similar enzymes. The readership will be benefitted from the Tables of global/tissue-specific knockdown (Table 1) and transgenic (Table 2) animal models of oxidative and antioxidant genes.

## Author contributions

MA and SA wrote the article and prepared the Tables of the genetically altered animals. MRA conceptualized the topic theme, edited the article and the tables, and wrote the abstract and the concluding remarks.

### Conflict of interest statement

The authors declare that the research was conducted in the absence of any commercial or financial relationships that could be construed as a potential conflict of interest.

## Key concepts

- **Paradoxical effects of ROS**: ROS may exert beneficial or detrimental effects on cardiovascular system depending on the levels, sub-cellular localization and duration 1281 of exposure to ROS.
- **Regulatable oxidant and antioxidant enzymes**: Genetically modified animals with on-off capability of the transgene.
- **Tissue-specific**: Tissue-specific (endothelium, cardiac, VSMC) knockout and transgene models
- **Sub-cellular ROS**: ROS in cytosol vs mitochondria vs. ER.

## Abbreviations

Ang-II: angiotensin-II
Akt: protein kinase B
AMPK: 5′ AMP-activated protein kinase
Atox1: antioxidant-1 copper chaperone
ATP: adenosine tri-phosphate
CaMKKb: calcium/calmodulin-dependent protein kinase kinase
cGMP: cyclic guanosine monophosphate
CVD, cardiovascular disease, CVS: cardiovascular system
EC: endothelial cell
eNOS: endothelial nitric oxide synthase
GPx: glutathione peroxidase
HO-1: heme oxygenase
iNOS: inducible nitric oxide synthase
KO: knockout
MAO: monoamine oxidase
MAPK: mitogen-activated protein kinase
MHEC: mouse heart endothelial cell
MMP: metalloproteinase
mTOR: mammalian target of rapamycin
NADPH: nicotinic adenine dinucleotide phosphate oxidase
NF-kB: nuclear factor kappa-light-chain-enhancer of activated B cells
nNOS: neuronal nitric oxide synthase
NO: nitric oxide
NOS: nitric oxide synthase
PI3K: phosphatidylinositol 3-kinase
ROS: reactive oxygen species
SDF1: stromal cell-derived factor 1
SOD: superoxide dismutase
Tet: tetracycline
Tg: transgene
Trx: thioredoxin
TrxR: thioredoxin reductase
VCAM-1: vascular cell adhesion molecule 1
VEGFR2: vascular endothelial growth factor receptor 2
VSMC: vascular smooth muscle cell
WT: wild-type
XO: xanthine oxidoreductase
BMT: bone marrow transplant.
